# Targeting immune checkpoint therapy: The role of manganese in tumor immunotherapy

**DOI:** 10.1016/j.cpt.2025.10.001

**Published:** 2025-10-24

**Authors:** Xingyao Lyu, Bixia Li, Zhijie Lin

**Affiliations:** a Department of Immunology, Faculty of Medicine, Yangzhou University, Yangzhou, Jiangsu 225009, China; b Key Laboratory of the Jiangsu Higher Education Institutions for Integrated Traditional Chinese and Western Medicine in Senile Diseases Control (Yangzhou University), Yangzhou, Jiangsu 225009, China

**Keywords:** Manganese, Immunotherapy, Immunogenic cell death, Tumor microenvironment

## Abstract

Cancer immunotherapy has emerged as a promising complement to traditional treatments such as radiotherapy and chemotherapy. Although conventional therapies remain central to cancer management, the potential of immunotherapy is increasingly recognized. Immune checkpoint therapy, a key strategy in tumor immunotherapy, has demonstrated significant efficacy against solid tumors. However, its clinical application is hindered by its limited response rate, necessitating efforts to optimize its effectiveness. Recent studies have highlighted the pivotal role of the cyclic GMP-AMP synthase (cGAS) - stimulator of interferon gene (STING) pathway in immune checkpoint therapy. Manganese (Mn), an essential trace element, regulates the activity of CD8^+^ T and natural killer (NK) cells by modulating the cGAS-STING pathway. Furthermore, the combination of Mn with anti-programmed cell death protein 1 therapy has demonstrated promising antitumor effects. Mn also influences immunogenic cell death (ICD), further augmenting its potential as an adjunct to tumor immunotherapy. Despite a growing body of research on the role of Mn in modulating the cGAS-STING pathway and inducing ICD, comprehensive reviews that synthesize these findings and explore the potential of Mn in enhancing immune checkpoint therapy are still lacking. This review aimed to fill this gap by examining the immune mechanisms by which Mn enhances immune checkpoint therapy and its overall impact on tumor immunotherapy.

## Highlights

• Mn^2+^ modulates the cyclic GMP-AMP synthase (cGAS) - stimulator of interferon gene (STING) pathway and promotes immunogenic cell death to enhance immune checkpoint therapy efficacy.
• Mn^2+^ binds to and activates cGAS, increasing its sensitivity to double-stranded DNA and promoting *cyclic GMP-AMP (*cGAMP) production.
• Manganese salts (e.g., MnJ) act as potent immune adjuvants that enhance both humoral and cellular immune responses.

## Graphical abstract

**Figure:**
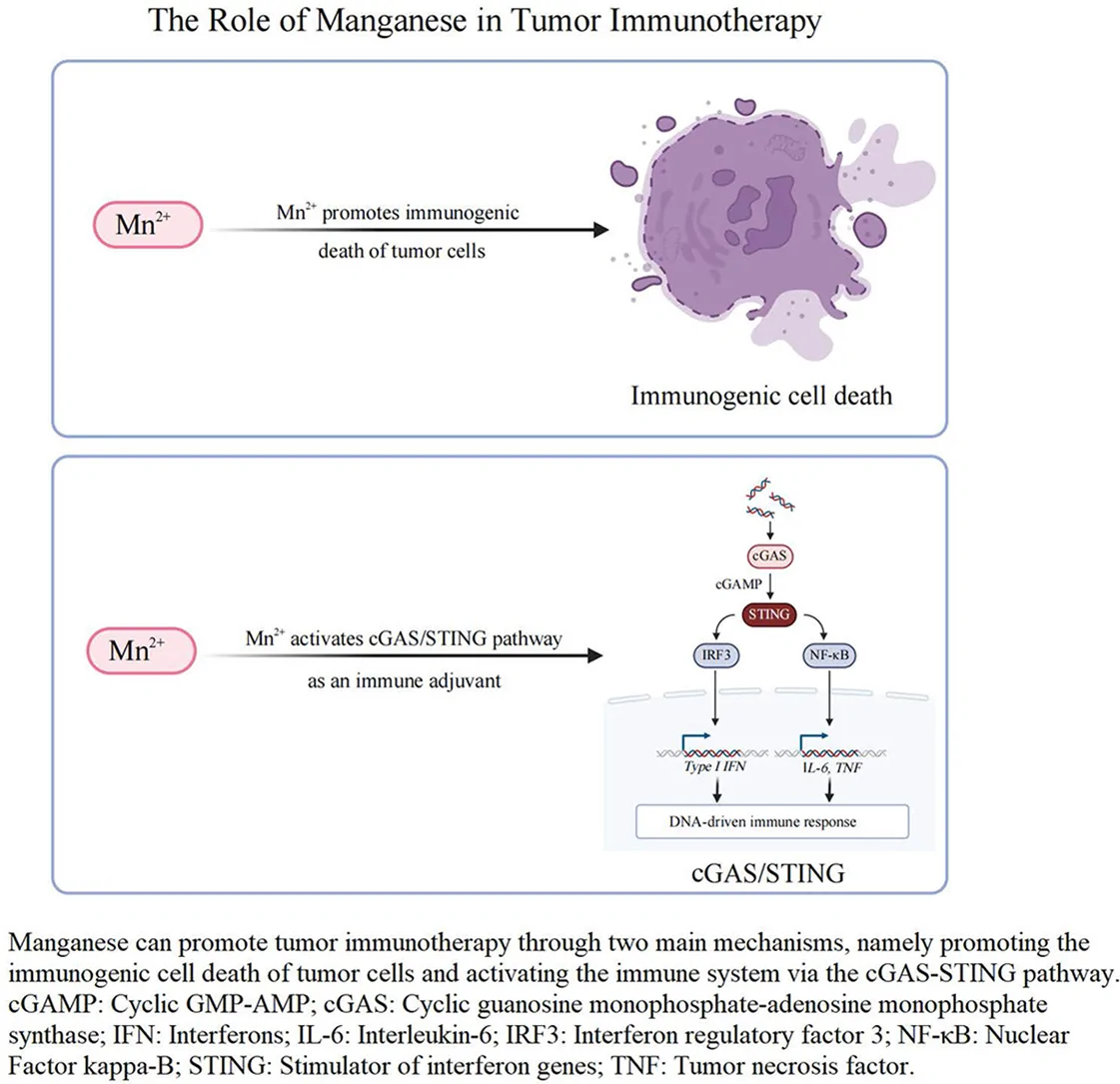

## Introduction

The introduction of immune checkpoint therapy has profoundly transformed traditional cancer treatment paradigms in recent years. Although radiotherapy and chemotherapy remain the dominant modalities for clinical tumor management, immune checkpoint therapy has shown significant therapeutic efficacy against solid tumors. This novel approach works by disabling the “brake” mechanism on T cells, thereby restoring their immune function and activating these cells to elicit antitumor responses. Since the U.S. Food and Drug Administration approved the first immune checkpoint inhibitor (ICI), an antibody targeting cytotoxic T-lymphocyte-associated protein 4, in 2011, the field of ICI therapy has expanded rapidly. Subsequent clinical advancements have focused primarily on targeting the T-cell co-inhibitory programmed cell death protein 1 (PD-1; also known as CD279) and its ligand, the programmed death-ligand 1 (PD-L1) signaling pathway. PD-1 is a co-inhibitory receptor that is expressed on the surface of antigen-stimulated T cells. PD-1 interacts with two ligands, PD-L1 (CD274) and PD-L2 (CD273).^1^ Clinical data demonstrate the high therapeutic efficacy of ICIs, positioning them as central to tumor immunotherapy research.^2^^,^^3^ However, only a small proportion of patients achieve a lasting response to monotherapy.^4^, ^5^, ^6^ This limited response may be attributed to insufficient T cell activation within the tumor microenvironment (TME). The limited therapeutic efficacy of ICIs in clinical practice is attributed to the characteristics of certain tumor types. Cold tumors, which are typically associated with low T-cell infiltration and an enhanced immunosuppressive microenvironment, illustrate this issue. This is evident in several solid tumors, including colorectal cancer,^7^ prostate cancer,^8^ and melanoma,^9^ where the therapeutic efficacy of ICI-based immunotherapy is limited. Hence, enhancing T-cell activation has become a crucial focus for improving the efficacy of ICI-based therapies and eliciting stronger antitumor immune responses.

Recent studies have demonstrated that mice deficient in the stimulator of interferon gene (STING) pathway fail to activate CD8^+^ T cells in various tumor therapy models.^10^, ^11^, ^12^ These findings suggest that activation of the cyclic GMP-AMP synthase (cGAS)-STING pathway is essential for promoting tumor-specific antigen presentation and CD8^+^ T cell activation, thereby enhancing the efficacy of anti-PD-1 or anti-PD-L1 therapies.^13^, ^14^, ^15^ Immunogenic cell death (ICD) influences the therapeutic efficacy of ICIs. ICD is a form of cell death that triggers an adaptive immune response against dying tumor cells. This response leads to the activation of antigen-specific T cells. ICD induction can enhance the effectiveness of immune checkpoint blockade by promoting the release of tumor-associated antigens and subsequent immune activation.^16^ Collectively, these findings underscore the importance of cGAS-STING pathway activation and ICD induction in mediating effective antitumor immune responses. The findings also highlight the critical roles of these pathways in the clinical success of immune checkpoint blockade therapies.

Manganese (Mn) is an essential trace element involved in various physiological processes, including enzyme activation and metabolism of glucose and lipids in humans.^17^ Importantly, Mn^2+^ enhances the sensitivity of cGAS to double-stranded DNA (dsDNA) and increases its enzymatic activity. Mn^2+^ also increases the binding affinity between *cyclic GMP-AMP (*cGAMP) and STING. Notably, Mn^2+^ is a potent activator of cGAS, inducing the production of interferons and cytokines in cells, even in the absence of infection.^18^ In addition to its role in the cGAS-STING pathway, Mn^2+^ also regulates ICD,^19^^,^^20^ contributing to its antitumor effects. Although numerous studies have demonstrated the regulatory role of Mn^2+^ in the cGAS-STING pathway and ICD, there is a lack of comprehensive reviews connecting these regulatory effects and summarizing the antitumor potential of Mn in targeting immune checkpoints.^20^ This review aimed to explore the specific immune mechanisms through which Mn modulates immune checkpoint therapy and its implications for tumor immunotherapy.

## Biological activity of manganese

Mn is involved in key functions, including development, reproduction, immune responses, metabolism, and antioxidant defense.^21^ Additionally, Mn forms complexes with several enzymes such as arginase, glutamine synthetase, phosphoenolpyruvate decarboxylase, pyruvate carboxylase, and Mn superoxide dismutase, highlighting its diverse biological functions.^22^ Given the importance of Mn in human health, maintaining adequate dietary intake is essential. Fortunately, Mn is readily available in various food sources, including legumes, rice, nuts, and whole grains. The National Research Council of the United States has established a recommended daily intake range of 2–5 mg for adults.^23^ The disruption of Mn homeostasis has been implicated in the pathogenesis of several neurological disorders.^24^ Although Mn deficiency is exceptionally rare in nonexperimental settings, studies on controlled dietary intake have linked insufficient Mn to impaired growth and poor bone development. Additionally, Mn deficiency is associated with abnormal glucose tolerance and altered lipid and carbohydrate metabolism. Moreover, below-average maternal blood Mn levels have been associated with low birth weight.^25^^,^^26^ Notably, the gut microbiota plays an important role in Mn metabolism.^27^ For example, after exposure to Mn, female mice showed an increase in Lactobacillus concentration, and this effect seemed to be related to sex differences in mice.^28^ Increased Mn transport genes in the gut microbiota of female mice have also been noted, suggesting that gut bacteria may subsequently produce Mn^2+^, thereby reducing its toxic effects by limiting Mn uptake into host cells.^29^ Hence, maintaining Mn homeostasis is vital for proper physiological function, and disturbances in this essential trace element have been implicated in the onset of various diseases, particularly those affecting the nervous system.

Mn toxicity or manganism is primarily associated with occupational and environmental exposure, particularly among miners, welders, and others who are frequently exposed to elevated Mn levels. Mn can cross the blood–brain barrier (BBB). Although the exact mechanism remains unclear, it is hypothesized to involve plasma ferroportin and transferrin.^30^ Manganism, a neurological disorder characterized by Parkinsonian-like symptoms, may arise from the Mn-induced alteration of BBB permeability, resulting in toxic effects of Mn and other harmful agents.^31^ The nervous system is the primary target of manganism, which manifests as cognitive, motor, and emotional impairments resembling those observed in Parkinson's disease.^32^ Individuals with liver failure or iron (Fe) deficiency, common global nutritional concerns, are particularly vulnerable to Mn toxicity. This vulnerability is because Mn is excreted via bile and competes with iron for similar transport mechanisms, which may lead to the accumulation of toxic levels over time.^33^^,^^34^ In recent years, Mn has attracted attention for its potential role in cancer therapy, particularly for its immunological effects in regulating immune checkpoints and its impact on CD8^+^ T cells within the field of cancer immunology.^35^ This emerging area of research underscores the need for further investigation into the antitumor properties of Mn and its influence on immune checkpoint pathways.

## Tumor immunotherapy based on Mn

### Mn in antitumor immunity through the cGAS-STING pathway

The cGAS-STING pathway is integral to the innate immune response, as it detects and responds to the presence of exogenous (from pathogens) or endogenous (from cellular damage) dsDNA. Upon binding to dsDNA, cGAS undergoes a conformational change that activates its catalytic function, leading to the production of the second messenger cGAMP. This step is crucial for initiating the cGAS-mediated immune signaling pathway. cGAMP then binds to and activates STING, which translocates from the endoplasmic reticulum (ER) to the Golgi apparatus. STING undergoes oligomerization and palmitoylation, facilitating the recruitment of TANK-binding kinase 1 (TBK1), which in turn activates interferon regulatory factor 3 (IRF3).^36^ The oligomerization of STING recruits and activates TBK1 dimers, leading to phosphorylation of the C-terminal domain of STING, which is essential for the activation of IRF3. Once activated, IRF3 moves to the nucleus, where it induces the expression of interferon-stimulatory genes (*ISGs*) and type I interferons (*IFNs*).^37^ IFNs, secreted by immune cells, exert paracrine effects on surrounding cells by binding the heterodimeric receptor interferon alpha receptor gene (*IFNαR*), composed of interferon Alpha/Beta Receptor 1 (IFNAR) 1 and IFNAR2 subunits coupled to the tyrosine kinases 2 and Janus Kinase 1 (JAK1), respectively.^38^ Receptor dimerization triggers JAK1 autophosphorylation, which in turn phosphorylates and activates signal transducer and activator of transcription (STAT) 1 and STAT2. These activated STATs associate with interferon regulatory factor 9 (IRF9) to form the transcription factor complex, IFN-stimulated gene factor 3 (ISGF3). ISGF3 translocates to the nucleus, binds IFN-stimulated response elements, and induces transcription of *ISGs*.^39^ Type I IFNs are central to the antiviral and immunomodulatory effects of the cGAS-STING pathway, underscoring their role in maintaining cellular homeostasis and defending against pathogens. This pathway is crucial for antiviral defense and plays a significant role in cancer immunotherapy by inducing immune responses through T cells and natural killer (NK) cells, as well as promoting the release of chemokines, pro-inflammatory cytokines, growth factors, and proteases that can lead to tumor cell senescence.^40^^,^^41^

In tumor immunotherapy, the cGAS-STING pathway plays a pivotal role in hindering tumor development. Cancer cells, which are often characterized by genomic instability, oxidative stress, and altered metabolism, are prone to DNA damage that can trigger the cGAS-STING pathway. This pathway not only activates an immune response but can also lead to cellular senescence. This process is characterized by the senescence-associated secretory phenotype, which involves the release of inflammatory mediators, proteases, and growth factors that suppress abnormal cell proliferation.^42^, ^43^, ^44^ Additionally, the cGAS-STING pathway can induce apoptosis in tumor cells by modulating the expression of pro-apoptotic and anti-apoptotic proteins,^45^ further contributing to tumor suppression. Importantly, external treatments such as chemotherapy and radiotherapy also induce DNA damage that can activate this pathway, leading to enhanced immune responses that contribute to tumor eradication.^14^

The involvement of Mn in modulating the cGAS-STING pathway offers promising insights into the enhancement of antitumor immunity. Studies have shown that Mn^2+^ (MnCl_2_-treatment *in vitro* and *in vivo*) enhances antiviral responses, with increased Mn^2+^ levels in cells, promoting cytokine production in response to DNA viruses. Contrastingly, similar to STING-null mice, Mn-deficient mice exhibit impaired cytokine production and are more susceptible to viral DNA infections. Notably, replenishing Mn^2+^ levels in Mn-deficient cells recovers their ability to respond to DNA viruses.^18^ In cancer therapy, Mn^2+^ (intravenously administered to tumor-bearing mice at 5 mg/kg) appeared to potentiate the cGAS-STING pathway by increasing the sensitivity of cGAS to dsDNA, enhancing its enzymatic activity, and promoting stronger STING activation. In Mn-deficient mice, tumor growth and metastasis were significantly accelerated, accompanied by a marked reduction in tumor-infiltrating CD8^+^ T cells, decreased antigen presentation, and impaired dendritic cell (DC) maturation. Mn^2+^ replenishment restores CD8^+^ T-cell activation and boosts NK cell activity. Notably, the Mn^2+^ -dependent cGAS-STING pathway promotes maturation of DCs and macrophages. This, in turn, strengthens the activation of CD8^+^ T and NK cells, leading to enhanced antitumor immune responses.^35^

In preclinical studies, Mn^2+^ has been shown to inhibit hepatocellular carcinoma progression in mice by enhancing immune regulation. Mn^2+^ treatment significantly reduced the number and weight of liver tumor nodules and increased the expression of type I IFN-stimulated genes, without directly affecting cytokine production by T cells. More importantly, Mn^2+^ enhances CD8^+^ T cell activity through other immune mechanisms, indicating that its antitumor effects are mediated through the activation of both type I IFNs and CD8^+^ T cells.^46^ Clinical exploration of STING agonists has demonstrated promising antitumor effects, but challenges remain owing to the metabolic instability of cyclic dinucleotide (CDN)-based STING agonists and their limited success in clinical trials.^47^ Currently, non-CDN STING agonists are being developed; however, their safety and efficacy are still under investigation.^48^, ^49^, ^50^ Nanoparticle-based drug delivery systems have emerged as potential solutions to enhance the stability and therapeutic effects of CDN-based STING agonists.^51^^,^^52^ Notably, when Mn^2+^ and CDN-based STING agonists are combined, they can self-assemble into coordination nanoparticles (CDN-Mn^2+^ particles, CMP), which demonstrate powerful antitumor immune effects upon local or systemic administration in preclinical models.^53^

Mn also enhances the effects of ICIs, such as anti-TGF-β/PD-L1 bispecific antibodies (YM101).^54^ TGF-β plays a critical role in immunosuppression by inhibiting T cell proliferation, promoting regulatory T cell differentiation, and impairing NK cell function.^55^ The development of YM101, which targets both TGF-β and PD-L1, has shown promising results in overcoming resistance to PD-1/PD-L1 therapies in high-TGF-β tumor models in preclinical studies. Combining Mn^2+^ *in vitro* with such bispecific antibodies could further improve antitumor efficacy by promoting the cGAS-STING pathway and regulating the immunosupportive TME.^56^

The inflammasome is a cytoplasmic multiprotein complex that is a significant component of the immune system.^57^ The Nod-like receptor 3 (NLRP3) inflammasome has garnered extensive research focus.^57^ Its structure consists of three core components: sensor NLRP3, adaptor protein apoptosis-associated speck-like protein containing a C-terminal caspase recruitment domain (CARD) (ASC), and the precursor enzyme pro-Caspase-1.^58^ Functioning as a detector for danger signals, the NLRP3 inflammasome responds to pathogen-associated molecular patterns (PAMPs) and damage-associated molecular patterns (DAMPs), leading to the maturation of pro-interleukin (IL)-1β and pro-IL-18.^58^ Under normal physiological conditions, intracellular concentrations of NLRP3, pro-IL-1β, and pro-IL-18 are maintained at minimal levels, ensuring a low inflammatory baseline.^59^ Detection of PAMPs or DAMPs by pattern recognition receptors (PRRs) initiates ‌nuclear factor kappa-B (*NF-κB*) nuclear translocation, which subsequently activates the transcription of genes encoding NLRP3, IL-1β, and IL-18.^59^ Activation involves the oligomerization of NLRP3, enabling its pyrin domain (PYD) to bind to the PYD of ASC. ASC then uses its CARD to engage the CARD of pro-Caspase-1, thereby assembling the active inflammasome complex. The NLRP3 inflammasome facilitates the auto-proteolytic cleavage of pro-Caspase-1 into its active form, Caspase-1. Caspase-1 possesses the critical ability to cleave gasdermin D. Meanwhile, Mn^2+^ can trigger NLRP3 inflammasome and induce cell pyroptosis via the caspase-1 pathway.^60^^,^^61^ One study introduced a multifunctional nanocube (Mn-ER-Cy) that combined MnCO_3_ and the ER-targeting photosensitizer. Nanocubes preferentially accumulate in tumor tissues and facilitate photothermal therapy, photodynamic therapy, and chemodynamic therapy (CDT) upon light irradiation. MnCO_3_ degrades into Mn^2+^ in an acidic TME, promoting reactive oxygen species (ROS) generation via a Fenton-like reaction. Triple-mode therapy simultaneously induces excessive ER stress, thereby activating the NLRP3 inflammasome, caspase-1, and gasdermin D pathways to enhance immunogenic pyroptotic cell death. Additionally, MnCO_3_ decomposition helps neutralize the acidic TME by consuming H^+^ and increasing the intracellular pH, supporting immune activation. Mn-ER-Cy also serves as a dual-mode imaging agent for near-infrared fluorescence and photoacoustic imaging, enabling precision-guided therapy.^62^ Additionally, one study designed a material in which tumor cells were engineered through layer-by-layer biomineralization, integrating silicification and Mn mineralization. This enhanced the mechanical stiffness of the cells to protect the antigens while creating spiky MnO_2_ nanoclusters. These clusters stimulate the NLRP3 inflammasome and cGAS-STING pathway to enhance immune responses. Biomineralized tumor cells outperformed monomineralic methods in preventing and treating the mouse B16F10 melanoma model.^63^

**Figure 1:**
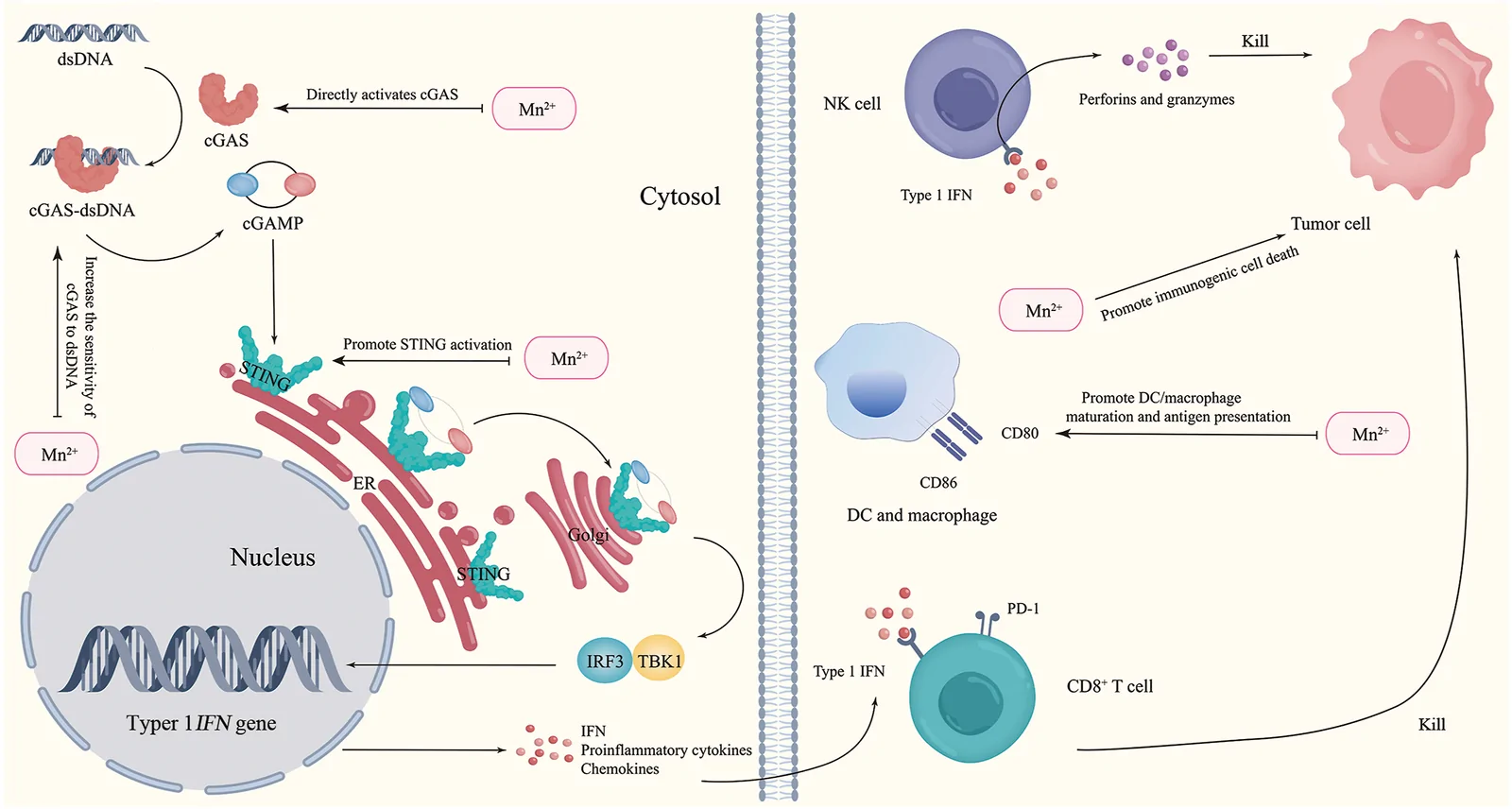
Schematic illustration summarizing the immunomodulatory effects of Mn²⁺ on the cGAS–STING pathway and tumor immunity. Mn^2+^ can directly activate cGAS, increase its sensitivity to dsDNA, and promote the generation of cGAMP. Mn^2+^ can also enhance the activation of STING and regulate the production of Type I interferons, thereby upregulating the effector function of CD8^+^T cells. Additionally, Mn^2+^ can promote antigen presentation by DCs and macrophages. cGAMP: Cyclic GMP-AMP; cGAS: Cyclic guanosine monophosphate-adenosine monophosphate synthase; DC: Dendritic cell; dsDNA: Double-stranded DNA; Mn^2+^: Manganese ion; STING: Stimulator of interferon genes.

In summary, Mn modulates the cGAS-STING pathway by enhancing the binding of dsDNA to cGAS, increasing its enzymatic activity, and improving STING activation. Through these mechanisms, Mn^2+^ plays a significant role in augmenting antitumor immunity and could be a valuable adjunct in cancer immunotherapy. Figure 1 summarizes the effects of Mn^2+^ on tumor immunity.

### Mechanisms of Mn regulation in ICD

ICD is a specialized form of regulated cell death that plays a pivotal role in activating the immune system, particularly in cancer.^64^ Although the immune system is highly effective at distinguishing “self” from “non-self,” certain disease models can inadvertently cause damage to the host's own tissues. Molecules released from dying cells or immune cells can serve as adjuvants or danger signals, collectively referred to as DAMPs.^65^ In the TME, DAMPs released by dying tumor cells are detected by innate PRRs, including Toll-like receptors (TLRs). This recognition triggers the activation of tumor-specific immune responses, frequently culminating in the formation of immune memory. In innate immune myeloid cells, TLRs induce the secretion of inflammatory cytokines, thereby engaging lymphocytes to mount an adaptive antigen-specific immune response that ultimately eradicates the invading microbes.^66^ Harnessing the potential of TLRs and related pathways has been applied to improve vaccine efficacy, treat cancers,^67^ inhibit their abnormal hyperactivity in inflammatory diseases,^68^ and modulate the immune response in autoimmune diseases such as systemic lupus erythematosus.^69^ Notably, accumulating evidence of ICD indicates that apoptotic caspase activity suppresses the production of type I interferons induced by mitochondrial DNA (mtDNA). When mtDNA is released into the cytoplasm, it activates the cGAS-STING pathway, thereby promoting an immune response.^70^^,^^71^

In recent years, various types of nanoparticles have gained increasing recognition for their ability to influence ICD, owing to the targeting capabilities of nanocarrier systems and their additional intrinsic advantages.^72^ Studies on nanomaterials have shown that these materials not only target tumor cells but also modulate the TME, overcoming immunosuppression, and thereby enhancing antitumor immunity.^73^ For instance, Fe_3_O_4_ and MnO_2_-based nanoparticles have been observed to reprogram tumor-associated macrophages (TAMs) from an immunosuppressive M2 phenotype to an antitumor M1 phenotype.^74^^,^^75^ Additionally, these nanoparticles can be combined with ICI therapy to improve the efficacy of immunotherapy.^76^^,^^77^ Specifically, Fe_3_O_4_ nanoparticles catalyze the conversion of hydrogen peroxide (H_2_O_2_) in a slightly acidic TME through CDT, producing toxic hydroxyl radicals that induce ICD. MnO_2_ nanoparticles exhibit TME-responsive glutathione (GSH)-depleting properties that enhance the CDT efficacy.^78^^,^^79^ When Fe_3_O_4_ and MnO_2_ nanoparticles are incorporated into a yolk-shell structure, they function as contrast agents, enabling dual-mode imaging, including magnetic resonance imaging (MRI) and diffusion tensor imaging with high accuracy and reliability. This yolk-shell architecture promotes extensive contact between the nanoparticles and H_2_O_2_ or GSH in the TME, thereby amplifying the CDT and ICD effects.^80^

Another promising nanomaterial, manganese zinc sulfide (ZMS) nanoparticles, has been effectively employed for melanoma treatment.^81^^,^^82^ ZMS nanoparticles generate substantial amounts of Mn^2+^-triggered hydroxyl radicals that contribute to a robust CDT.^83^^,^^84^ The application of PPIR780-labeled ZMS micelles has been shown to inhibit primary melanoma growth, reduce lung metastasis, and improve the survival of mice with B16F10 melanoma.^19^ Figure 1 summarizes the effects of Mn^2+^ on tumor immunity.

## Clinical application of Mn in tumor immunotherapy

### The clinical toxicity of Mn-based nanomaterials

Using manganese-enhanced MRI (MEMRI) technology, studies of superoxide dismutase 2 (*SOD2*) gene polymorphisms in breast cancer clinical trials and phase I studies of manganese superoxide dismutase (MnSOD) plasmid liposomes in patients with non-small-cell lung cancer have investigated the biomedical applications and potential toxicity of Mn. MEMRI-based research revealed that Mn signals under physiological and pathological conditions can reflect neural activity and excitotoxic events. These findings laid the foundation for its application in neuroimaging.^85^ One study indicated that *SOD2* gene polymorphisms are associated with treatment-related toxicity and disease-free survival in breast cancer, where the C allele, linked to higher antioxidant activity, may reduce treatment-induced toxicity and shorten disease-free survival.^86^ A phase I clinical trial showed that the oral administration of MnSOD plasmid liposomes is feasible and safe, with no dose-limiting toxicities reported at a dose of 30 mg.^87^ To optimize the clinical application of Mn, future research should focus on refining the dosing regimens, including route and frequency adjustments, to minimize its effect in normal tissues. The development of novel Mn-based nanomaterials with targeted delivery and controlled release capabilities is also promising and can enhance site-specific delivery and reduce off-target effects. These approaches can preserve the therapeutic efficacy of Mn while lowering the risk of toxicity, thereby facilitating its broader clinical use.

### Mn acts as an immune adjuvant

Vaccination remains one of the most effective strategies for addressing public health crises. Vaccine adjuvants play a pivotal role in enhancing vaccine efficacy by accelerating and amplifying immune responses, causing faster and more robust protection. Adjuvants are generally classified into two categories: immunoenhancers, which boost the immune response, and delivery systems, which facilitate antigen delivery.^88^^,^^89^

In the 1920s, McKee et al. first discovered the immune-enhancing properties of aluminum salts (Alum).^90^ Since then, aluminum-containing adjuvants have been widely used in vaccines, primarily eliciting Th2 responses but showing limited efficacy in stimulating Th1 or cytotoxic T lymphocyte (CTL) responses. Since the introduction of aluminum-based adjuvants, additional metal salts have been explored for their immunomodulatory effects.^91^^,^^92^ Among these, Mn^2+^ has been shown to enhance immune responses by promoting antigen uptake, antigen presentation, and germinal center formation, which is mediated through the activation of the cGAS-STING and NLRP3-ASC pathways.^93^

A notable advancement in this area is the development of a colloidal Mn salt, colloidal manganese salt adjuvant (MnJ), which has demonstrated broad-spectrum adjuvant activity. MnJ induces both humoral and cellular immune responses, primarily by enhancing CTL activation. Moreover, when administered intranasally, MnJ acts as a mucosal adjuvant and stimulates the production of high levels of secretory immunoglobulin A. This adjuvant exhibits potent effects on various antigens, including T cell-independent antigens, making it a promising candidate for future vaccine formulations.^92^

### The role of Mn as a nanomaterial in tumor immunotherapy

Traditional cancer therapies, particularly chemotherapy, are often associated with significant toxicity and limited targeting. Although chemotherapeutic agents can induce ICD, their clinical application is often hindered by their poor tolerability and suboptimal ICD induction.^94^^,^^95^ This has spurred the development of effective ICD inducers.

One such emerging candidate is ZMS nanomaterial, which was previously highlighted as a novel ICD inducer. Additionally, manganese oxide (MnOx) nanotips have shown promise as nanoadjuvants and ICD-inducing agents targeting the TME. These nanotips have been employed in magnetic resonance and photoacoustic image-guided immunotherapies using cancer nanovaccines. The cytotoxic effects of MnOx nanotips are primarily attributed to the dual actions of Mn^2+^-mediated CDT and GSH-depleting ferroptosis. Both CDT and ferroptosis contribute to ICD by triggering the release of DAMPs from dying tumor cells. Hence, MnOx nanotips, similar to MnO_2_ nanoparticles, serve as potent ICD inducers, offering a promising approach to enhance the efficacy of cancer immunotherapy.^96^ In a phase I clinical trial of Mn, blood manganese concentration in cancer patients was within the normal range (0.029–1.200 μmol/L), serving as a clinical indicator to measure whether the therapeutic dose of Mn administered is beyond the therapeutic range and thus toxic to the patient.^35^ In a clinical study, Avasopasem Mn (AVA) was used to treat renal injury after radiotherapy in patients with oral or oropharyngeal squamous cell carcinomas. Patients were randomized to receive a placebo, 30 mg AVA, or 90 mg AVA. Treatment with 90 mg AVA, compared to 30 mg AVA and placebo, prevented a significant reduction in estimated glomerular filtration rate (eGFR) at 3, 6, and 12 months after treatment, and no patient-specific toxicities were identified.^97^

### The potential of combination therapy

Mn-based nanomaterials exhibit significant synergistic effects when combined with chemotherapy by enhancing drug delivery and modulating the TME. Manganese oxides (e.g., MnO_2_) can serve as efficient drug carriers. Their unique pore structure and surface properties enable high drug loading and slow release, which prolongs drug circulation in the body and increases drug concentration within the tumor tissue, thereby enhancing efficacy while reducing side effects. For instance, hollow mesoporous MnO_2_ nanostructures loaded with doxorubicin can precisely release the drug in the TME, with the release triggered by acidity and high GSH levels.^98^ This approach effectively inhibits tumor growth while minimizing systemic toxicity. Mn-based materials can also modulate the TME to enhance chemotherapeutic drug sensitivity. MnO_2_ nanosheets, which decompose H_2_O_2_ to generate oxygen and consume GSH, alleviate TME hypoxia and increase ROS levels, making tumor cells more susceptible to chemotherapeutic agents.^98^^,^^99^ Furthermore, manganese ions (Mn^2+^) can activate the cGAS-STING signaling pathway, boost antitumor immune responses, and synergistically enhance the overall therapeutic outcome.^35^^,^^100^ For example, Mn-doped mesoporous silica nanoparticles loaded with immunostimulants can achieve synergistic chemoimmunotherapy, effectively inhibiting tumor growth and preventing recurrence.

In addition to chemotherapy, many Mn-based nanomaterials can generate ROS and increase oxygen supply at tumor sites, thereby alleviating tumor hypoxia and enhancing radiotherapy efficacy.^101^ For example, MnO_2_ nanoparticles react with H_2_O_2_ in the TME to produce O_2_, improve the oxygen supply, and increase tumor cell sensitivity to radiotherapy.^102^ Radiotherapy damages tumor cell DNA by generating ROS, and Mn-based nanomaterials can further catalyze ROS production to enhance this effect.^103^ Some Mn-based nanomaterials, such as those containing Mn^3+^ and Mn^4+^, have Fenton-like activity, converting H_2_O_2_ into highly oxidative ·OH radicals to increase tumor cell oxidative damage and enhance radiotherapy cytotoxicity.^104^ Additionally, since high intracellular GSH levels in tumor cells can scavenge ROS and reduce radiotherapy efficacy, Mn-based nanosystems can oxidize GSH to glutathione disulfide‌, thus lowering GSH levels in the TME.^105^^,^^106^ This prolongs ROS activity, thereby potentiating radiotherapy-induced tumor cell damage. The manganese clinical trials are summarized in Table 1.

**Table 1:** Ongoing or completed clinical trials evaluating manganese-based interventions in tumor immunotherapy (Data obtained from https://clinicaltrials.gov/).

NCT number	Trial phase	Manganese formulation	Intervention plan	Patient population
NCT04873440	Phase I/II	MnCl_2_	Manganese plus radiotherapy	Solid tumors or lymphoma
NCT03991559	Phase I	MnCl_2_	Manganese plus anti-PD-1 antibody	Solid tumors or lymphoma
NCT03989336	Phase II	MnCl_2_	Manganese primed sintilimab plus nPP chemotherapy	Ovarian cancer
NCT03989310	Phase I/II	MnCl_2_	Manganese primed anti-PD-1 antibody plus nPG chemotherapy	Local advanced/metastatic pancreatic cancer
NCT04004234	Phase I/II	MnCl_2_	Manganese primed anti-PD-1 antibody plus nPG chemotherapy	Local advanced/metastatic biliary tract cancer
NCT05131711	Phase I/II	MnCl_2_	Stereotactic radiosurgery plus immunal adjuvants (GM-CSF, sapylin, MnCl_2_)	Recurrent glioblastoma

GM-CSF: Granulocyte-macrophage colony-stimulating factor; nPG: Nanoparticle albumin-bound paclitaxel and platinum; nPP: Nanoparticle albumin-bound paclitaxel-gemcitabine; PD-1: Programmed cell death protein 1.

## Discussion

Traditional cancer therapies such as radiotherapy and chemotherapy often face significant challenges, including poor targeting, high toxicity, and drug resistance. Contrastingly, metal ion therapy can effectively kill tumor cells while minimizing adverse reactions and reducing drug resistance. Recent studies have highlighted the potential of iron therapy in modulating the TME.^107^^,^^108^ Tissue-resident macrophages are influenced by intracellular iron metabolism, leading to altered macrophage polarization in response to various microenvironmental stimuli.^109^^,^^110^ The infiltration of iron-loaded TAMs has been correlated with tumor regression in patients with non-small cell lung cancer, suggesting that targeted iron delivery to TAMs could serve as an adjuvant therapeutic strategy to enhance antitumor immune responses.^111^, ^112^, ^113^ Additionally, iron metabolism affects the activity of CD8^+^ T cells,^114^ while zinc, another essential element, influences immune cell activity.^115^ As a known tumor immune adjuvant, the ability of Mn to form composite materials with other metals to enhance the effect of tumor immunotherapy is currently being investigated. Mn galvanic cells are prepared via liquid-phase exfoliation and *in situ* galvanic replacement to modulate tumor metabolism, thereby enhancing cGAS-STING activation for bidirectional synergistic H_2_-immunotherapy.^116^

Several Mn-based nanodelivery systems have been investigated for use in cancer immunotherapy. Mn^2+^-anchored mannose-modified bovine serum albumin (BSA) and β-lapachone-loaded ferritins are crosslinked to promote bioresponsive protein nanoassemblies, which dissociate into monodispersive protein units in acidic perivascular TME, thus enabling enhanced tumor penetration and spatiotemporally controlled Mn^2+^ and β-lapachone delivery to DCs and tumor cells, respectively. β-lapachone causes immunogenic tumor cell apoptosis and releases abundant dsDNA into TME, while Mn^2+^ enhances the sensitivity of cGAS to dsDNA and augments STING signaling to trigger downstream immunostimulatory signals.^117^

SOD2 plays a pivotal role in tumor and immune regulation.^118^ *SOD2* overexpression enhances cell invasiveness and migration through different mechanisms.^119^ Mn-dependent SOD is specifically located in the mitochondria and scavenges superoxide radicals directly formed in the respiratory electron transport chain.^120^^,^^121^ Its modulation alters the amount of H_2_O_2,_ a stable ROS messenger, with important consequences in cell cycle signaling, and thus in cancer.^122^ Therefore, this represents a promising future direction for elucidating the role of Mn in tumor immunity. Whether the genetic epigenetics of SOD2 can also contribute to the efficacy of Mn should be explored.

However, in the clinical application of Mn, we have to consider an important issue: the dual effect of Mn on tumors. Excessive use of Mn may lead to neurotoxicity in patients, necessitating the need for safe use of Mn to prevent neurotoxicity in patients. Therefore, we have listed the concentration of Mn for clinical treatment and its physiological effect, providing researchers with a reference. At the same time, there are many studies on how to alleviate the neurotoxicity caused by overdose.^123^^,^^124^ When Mn acts as an immunostimulant to enhance the host immune system and improve the antitumor ability, it may also lead to the generation and enhancement of inflammation. This inflammatory response may lead to the occurrence of tumors, such as colitis-related colon cancer. Therefore, these issues must be investigated.

The potential use of Mn in tumor immunotherapy opens new avenues for research. Furthermore, future studies should explore the regulatory effects of metals, such as iron and zinc, on immune cells, as well as the influence of Mn on additional cell signaling pathways in cancer immunotherapy. Additionally, there is a need to investigate the role of Mn in the TME (particularly its ability to reverse immunosuppression in tumors) and delve deeper into its underlying mechanisms.

### Limitations of the review

Despite the promising evidence outlined herein, some limitations of the review must be acknowledged. First, most mechanistic insights have originated from preclinical mouse models or *in vitro* studies. The immune microenvironment, pharmacokinetics, and STING genetics profiles of these systems differ substantially from those of humans; therefore, the direct translation of Mn-based interventions remains uncertain. Second, the therapeutic window for systemic Mn^2+^ administration is narrow. Excessive accumulation causes neurotoxicity and cardiotoxicity, which may be mitigated by nanotechnology-based strategies (e.g., nanoparticle-mediated targeted delivery) to reduce off-target damage to the nervous system and other tissues. However, the dose-response relationships for antitumor efficacy vs systemic toxicity have not been systematically mapped in large animals or humans. Third, most studies employed supraphysiological Mn concentrations or Mn nanomaterial formulations that may behave very differently from dietary or parenteral Mn salts. Consequently, the relevance of these findings to clinically achievable Mn levels remains unclear. Fourth, the review is limited by the scarcity of completed clinical trials; only early-phase safety data for STING agonists with or without Mn are available, and randomized evidence evaluating Mn as an adjuvant to immune checkpoint blockade is still lacking. Fifth, heterogeneity in tumor genotypes, microbiome composition, baseline Mn status, and prior therapies could modulate responsiveness to Mn, though patient stratification biomarkers have not yet been defined. Finally, the potential interactions between Mn and standard-of-care agents (e.g., platinum drugs) or comorbidities (iron overload and liver dysfunction) remain underexplored. These gaps highlight the urgent need for rigorously controlled clinical trials. Such studies should include pharmacokinetic monitoring, biomarker validation, and long-term safety assessments before Mn-based strategies are widely adopted in oncology.

## Authors contribution

Xingyao Lyu and Bixia Li were responsible for drafting the manuscript, preparing and revising figures; Zhijie Lin provided critical revisions to the manuscript, contributing significant intellectual content. All authors reviewed and approved the final version of the manuscript.

## Ethics statement

None.

## Data availability statement

The datasets used in the current study are available from the corresponding author on reasonable request.

## Declaration of Generative AI and AI-assisted technologies in the writing process

The authors declare that generative artificial intelligence (AI) and AI assisted technologies were not used in the writing process or any other process during the preparation of this manuscript.

## Funding

This study was supported by the National Natural Science Foundation of China (No. 82102901) and the Innovation and Entrepreneurship Training Program for College Students (No. 202311117044Z) of Jiangsu Province, China.

## Conflict of interest

The authors declare that they have no known competing financial interests or personal relationships that could have appeared to influence the work reported in this paper.
